# 

**Published:** 1886-03

**Authors:** 


					﻿IMAJRCJH:, 18863-
OLD SERIES
v’'-w '
SERIES
IW?IK.-No. I.
& 1855
Mm®
HEBIGIL^WRMs
Jnnsio
I et	’ lore •)
Wc&ic^-vl"- S).
&«%
W.F.WESTMORELAND. lO
H.V.M.MILLER.M.D.LL .D.®
JAMES A.GRAY, MD.
MihUSHED BY JAMES P.HARRISON&.CGS
W	Atlanta,ga.
SUBSCRIPTION: $2.50 A YEAR.
				

